# Transcriptome analysis reveals the mechanism for blue-light–induced biosynthesis of delphinidin derivatives in harvested purple pepper fruit

**DOI:** 10.3389/fpls.2023.1289120

**Published:** 2023-10-26

**Authors:** Jinhui Gao, Yuwei Dou, Xiaotong Wang, Dalong Zhang, Min Wei, Yan Li

**Affiliations:** ^1^ College of Horticultural Science and Engineering, Shandong Agricultural University, Tai’an, Shandong, China; ^2^ Scientific Observing and Experimental Station of Environment Controlled Agricultural Engineering in Huang-Huai-Hai Region, Ministry of Agriculture, Tai’an, Shandong, China

**Keywords:** light spectrum, blue light, purple pepper, delphinidin derivatives, transcriptomic

## Abstract

Anthocyanins are the main pigments that affect the color and quality of purple-fruited sweet pepper (*Capsicum annuum*). Our previous study indicated that blue light can induce anthocyanin accumulation in purple pepper. In view of its underlying mechanism that is unclear, here, anthocyanin content was determined, and transcriptome analysis was performed on pepper fruits harvested from different light treatments. As a result, among the identified anthocyanin metabolites, the levels of delphinidin (Dp) glycosides, including Dp-3-*O*-rhamnoside, Dp-3-*O*-rutinoside, and Dp-3-*O*-glucoside, were highly accumulated in blue-light–treated fruit, which are mainly responsible for the appearance color of purple pepper. Correlation between anthocyanin content and transcriptomic analysis indicated a total of 1,619 upregulated genes were found, of which six structural and 12 transcription factor (TF) genes were involved in the anthocyanin biosynthetic pathway. Structural gene, for instance, *CaUFGT* as well as TFs such as *CaMYC2*-*like* and *CaERF113*, which were highly expressed under blue light and presented similar expression patterns consistent with Dp glycoside accumulation, may be candidate genes for anthocyanin synthesis in response to blue-light signal.

## 1 Introduction

Peppers (*Capsicum annuum* L.) are one of the world’s most important vegetables and are favored by consumers for their vibrant color and nutritional benefits. The color of pepper fruit is influenced by the pigment content of chlorophylls, carotenoids, anthocyanins, etc., (Liu et al., 2022b). Among them, anthocyanins not only are an important factor in promoting fruit coloration but also have a rich nutritional and medicinal value (Liu et al., 2018b; Colanero et al., 2020; Sun et al., 2023). The different color-fruited genotypes of pepper have shown variation in kinds and content of anthocyanins (Lightbourn et al., 2007; Tang et al., 2020; Guo et al., 2021), among which delphinidin (Dp) derivatives were found to make a major contribution to the purple color formation of unripe pepper fruit in our previous study (Wang et al., 2022b).

Anthocyanin is the final product of flavonoids pathway, the biosynthesis of this metabolite has been clarified in many crops, and it was also well studied in pepper (Aguilar-Barragán and Ochoa-Alejo, 2014; Liu et al., 2018b; Meng et al., 2022; Xie et al., 2022). The structural genes associated with the anthocyanin biosynthetic pathway are usually divided into early (EBGs) and late biosynthetic genes (LBGs). Of the latter cluster, flavonoid 3′,5′ hydroxylase (*F3′5′H*), dihydroflavonol 4-reductase (*DFR*), anthocyanidin synthase (*ANS*), and uridine 5′-diphosphate (UDP)-glucose: flavonoid 3-O-glucosyltransferase (*UFGT*) are essential for producing Dp glycosides, which determines the purple color (Tanaka and Brugliera, 2013; Naing and Kim, 2018). In addition, previous studies indicated that the transcription of LBGs is activated by several transcription factors (TFs), including NAM-ATAF1/2-CUC2 (NAC), basic leucine zipper (bZIP), and ethylene-responsive factor (ERF) families, along with MYB-bHLH(basic helix-loop-helix)-WD40 (WD40-repeat proteins) (MBW) complex, within which the activation or repression is mainly determined by v-myb avian myeloblastosis viral oncogene homolog (MYB) (Liu et al., 2016; Liu et al., 2018b).

Light is one of the most critical environmental factors regulating anthocyanin biosynthetic pathway (Guo et al., 2022; Li et al., 2023). In addition to light intensity, light spectrum, especially blue light, has been demonstrated to regulate anthocyanin accumulation (Liu et al., 2022b; Lee and Kim, 2023). For instance, the elevated anthocyanin accumulation was found concurrently when exposed to blue light and overexpressed *CRY1a* in tomato fruit (Liu et al., 2018a), and the anthocyanin levels from postharvest purple pepper fruit were enhanced with a higher fraction of blue light (Liu et al., 2022b). Moreover, blue light could induce the expression of downstream anthocyanin synthesis structural genes through cryptochrome (CRY)-constitutive photomorphogenesis protein 1 (COP1)-MYB and/or -elongated hypocotyl 5 (HY5), which, in turn, accelerating anthocyanin accumulation in eggplant (Jiang et al., 2016), red pear (Tao et al., 2018), and petunia petals (Fu et al., 2020).

Anthocyanin accumulation is essential for the fruit quality of purple pepper, and our previous study showed that blue light could promote anthocyanin synthesis more than white light in purple pepper (Wang et al., 2022b). Although multiple TF and structural genes involved in anthocyanin biosynthesis have been well studied, the molecular mechanism of blue light regulating anthocyanin synthesis in purple pepper remains unclear. In the present study, we thus performed transcriptome analysis to characterize the potential transcripts involved in the anthocyanin accumulation in postharvest pepper fruit irradiated under blue light. These findings may expand our understanding of the molecular mechanism of blue light in regulating pepper anthocyanin biosynthesis.

## 2 Materials and methods

### 2.1 Plant materials and experimental conditions

**Figure 1:**
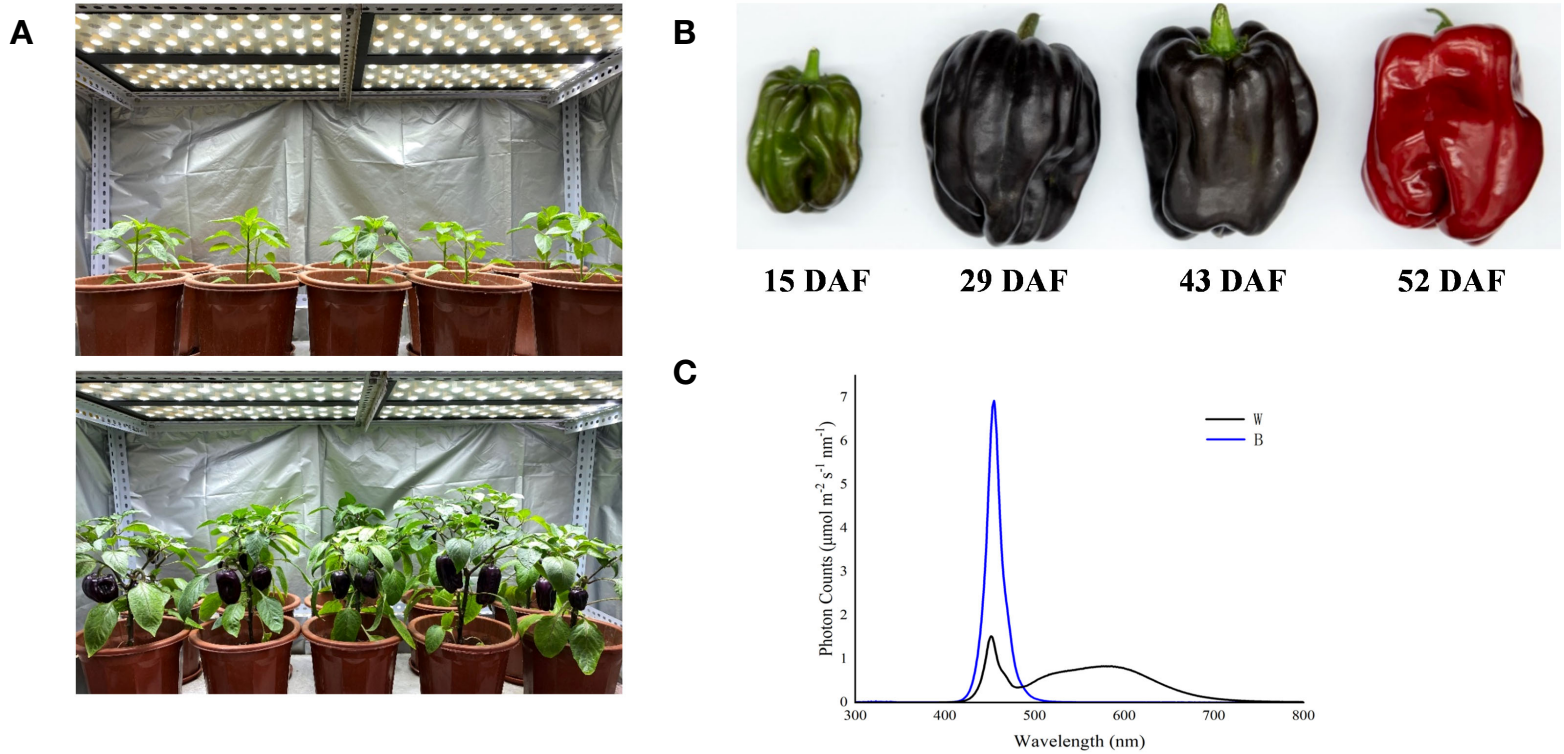
Spectral light treatments of purple pepper plants and fruits. **(A)** Purple pepper plants grown under white light before treatment. **(B)** Developmental stages of pepper fruit: 15 DAF, small unripe green fruit; 29 DAF, large unripe purple fruit; 43 DAF, ripening purple fruit; 52 DAF, fully ripening red fruit. **(C)** Spectral distribution of light treatments irradiated on purple pepper fruits. DAF, day after flowering; W, white light; B, blue light.

The experiment was performed from April to September 2021, and the purple-fruited pepper cultivar (*Capsicum annuum* cv. Amethyst) was planted in a growth chamber, where the average air temperature, relative humidity, photoperiod, and CO_2_ concentration is 400 mmol mol-1 were 26°C/18°C (day/night), 70%, 14 h/10 h, respectively. The pepper plants were irradiated with white light LED at the photosynthetic photon flux density (PPFD) of 200 μmol m^−2^ s^−1^ (**Figure 1A**). In our previous study, the total content of anthocyanins and expression levels of key structure genes involved in the anthocyanin biosynthesis were determined in pepper fruits, which harvested at different ripening stages of 15, 29, 43, and 52 days after flowering (DAF; **Figure 1B**) and then irradiated under white and blue light for 4 h. These parameters were found to be enhanced significantly at 29 DAF (Wang et al., 2022b). Therefore, in this study, the defect-free fruits with similar size and setting position were harvested at 29 DAF and randomly divided into two groups. Subsequently, all fruits were transferred and irradiated under blue (457.2 nm) and white (control) LED light sources (**Figure 1C**) with a PPFD of 200 μmol m^−2^ s^−1^, and the peel was subsequently collected at different treatment duration of 0 h, 2 h, 4 h, 8 h, 12 h, and 24 h with three replicates (at least three fruits for each replicate per sampling stage). Fruit peels were manually separated by using surgical scalpel blades, avoiding as much as possible to collect flesh material. Each peel sample was constituted of 2-cm-wide and 1-mm-thick skin taken from the equatorial part, then immediately frozen in liquid nitrogen, and stored at −80°C.

### 2.2 Determination of anthocyanin content

In our previous study, the metabolite profiling of pepper fruits at different ripening stages of 15, 29, 43, and 52 DAF irradiated by white and blue spectrum for 4 h was performed using a targeted metabolome method by Wuhan Metware Biotechnology Co., Ltd. (Wuhan, China), and most anthocyanins were found to be enriched at 29 DAF. In the present study, pepper peels were thus sampled from fruit at 29 DAF with different treatment duration of 0 h, 2 h, 4 h, 8 h, 12 h, and 24 h as well as with 4 h of white and blue-light treatment for determination of total anthocyanin content and quantification of anthocyanins, respectively. To measure total anthocyanins according to Liu et al. (2022b) with some modifications, each 0.5-g peel tissue sample was combined with 0.5% hydrochloric acid methanol reagent, vortexed, and incubated at 40°C for 40 min. Then, the samples were centrifuged at 10,000 g for 5 min at 4°C, and the supernatants were collected. The absorbance of samples was measured at 530 nm, 620 nm, and 650 nm, respectively, using an ultraviolet-visible spectrophotometer (UV-5500, Bilon Instrument Co., Ltd., Shanghai, China). Total anthocyanin content was calculated according to the following formula: (1) △OD = (OD_530_ − OD_620_) − 0.1 × (OD_650_ − OD_620_) and (2) anthocyanin content = (△OD × V × 10^6^) / (ξ × m), where V is the dilution volume, ξ is the molar absorption coefficient, and m is the fresh weight of sample.

To quantify the most abundant anthocyanin, the freeze-dried samples were crushed, and 50 mg of powdered tissue was extracted overnight at 4°C in 500 μL of 50% aqueous methanol. Following centrifugation at 12,000 g under 4°C for 3 min. Then, these extracts were absorbed, filtrated, and analyzed by an ultra-performance liquid chromatography with tandem mass spectrometry (UPLC-MS/MS. UPLC, Shim-pack UFLC SHIMADZU CBM30A, Shimadzu Scientific Instruments, Inc., Kyoto, Japan; MS, Applied Biosystems 6500 Triple Quadrupole, AB SCIEX, Framingham, USA) system. Analytical conditions were as follows: HPLC column, Waters ACQUITY BEH C18 (1.7 μm, 2.1 mm × 100 mm, Milford, USA). The mobile phase comprised solvent A (ultrapure water, 0.1% formic acid) and solvent B (methanol, 0.1% formic acid). Gradient programs were as follows: solvent A:solvent B (v:v), 95:5 at 0 min, 50:50 at 6 min, 5:95 at 12 min, maintained at 2 min, 95:5 at 14 min, and maintained at 2 min. The flow rate, column temperature, and injection volume were set as 0.35 mL/min, 40°C, and 2 µL, respectively. The effluent was alternatively connected to the Q-Trap 6500 equipped with an electrospray ionization (ESI) according to Liu et al. (2020). The MS parameters mainly including 550°C of ESI source temperature, 5,500 V for ion spray voltage floating, and 35 psi of curtain gas. In Q-Trap 6500, each ion pair was scanned on the basis of the optimized declustering potential and collision energy.

### 2.3 Expression of anthocyanin biosynthetic genes

Total RNA was extracted from fruit harvested at 29 DAF with treatment duration of 0 h, 2 h, 4 h, 8 h, 12 h, and 24 h under white and blue light by the Quick RNA Isolation Kit (Huayueyang Biological Technology Co., Ltd., Beijing, China). Reverse transcription was performed with the ReverTra Ace qPCR RT-Kit (Toyobo Bio-Tech, Co., Ltd., Toyobo, Japan). Expression of structural genes involved in the anthocyanin biosynthesis was conducted by employing quantitative real-time PCR (qRT-PCR) under the following programs: 95°C for 30 s, followed by 40 cycles of 95°C for 15 s, 60°C for 30 s, and 95°C for 15 s. The relative expression levels of the genes were analyzed by the 2^−ΔΔCt^ method (Livak and Schmittgen, 2001). The information of the primers used for the qRT-PCR is given in **Table 1**.

**Table 1:** Sequences of primers for RT-qPCR.

Gene	Forward primer sequence (5′-3′)	Reverse primer sequence (5′-3′)	Gene ID
*CaCHI*	CCTTGCTGGTGCAGGGATTA	GGAACGGCACTCTCTTCCAT	LOC107871144
*CaF3H*	AGGCAGTAATGGATGAGCCC	CTCAATGGGCATGGATTCCAAC	LOC107859880
*CaF3′5′H*	GGCCTACAATGCCCAAGACA	ATATCCGCCACCACAACGC	LOC107848667
*CaDFR*	CGGCTGGATTTATCGGCTCT	CTTCCACGGTCAAGTCTGCT	LOC107860031
*CaANS*	TTCTCCTCCCAGACACCGAT	AATCACTCTGTGCTCCACGC	LOC107843451
*CaUFGT*	AAACAAGGCAATGACACCCC	TTCCTCCTCTGCCTCTTTCA	LOC107867844
*CaMYB1R1*	ATGGACGGAGGTGGAGC	GGCGACGATTGAGGTTAGT	LOC107862462
*CabHLH13*	AATGTGAAGCCAGTGAA	ACAACAGCTCGTAATGC	LOC107854119
*CaERF113*	AGGGAGCGGGAGAAGAC	TGGGCATACTGGAGAAGGT	LOC107858557
*CaMYC2-like*	AGAAAGGCAAAAGCAGCAGG	TGGTTCAAAGGCGTTTCACG	LOC107845814
*Actin*	GTCCTCTTCCAACCATCCAT	TACTTTCTCTCTGGTGGTGC	LOC107850541

### 2.4 Transcriptome sequencing and data analysis

Transcriptome sequencing and Library preparation were conducted by Wuhan Metware Biotechnology Co., Ltd. (Wuhan, China). Total RNA was extracted from the same peel powder, which collected from fruits at 29 DAF irradiated by 0 h, 2 h, and 4 h of white and blue light, to construct an mRNA library, and sequenced on the Illumina HiSeq 4000 platform. After raw data filtering, sequencing error rate checking, and guanine-cytosine (GC) content distribution detection, clean reads for subsequent analysis were obtained, and, then, the clean reads were mapped to the pepper reference genome (Zunlal v1.0) using HISAT2 (Kim et al., 2015). On the basis of the raw data, screening for differentially expressed genes (DEGs) was performed using DESeq2 software. Gene expression levels were assessed on the basis of the fragments per kilobase of transcript per million fragments mapped reads (FPKM) method. Genes meeting the threshold of |log_2_fold change| ≥ 1 and false discovery rate < 0.05 were defined as the DEGs and were subjected to Gene Ontology (GO) and Kyoto Encyclopedia of Genes and Genomes (KEGG) enrichment analysis.

### 2.5 Validation of DEGs by qRT-PCR

To validate the results obtained from transcriptome sequencing, qRT-PCR analysis was performed. Samples were collected from fruits at 29 DAF irradiated by 0 h, 2 h, and 4 h of white and blue light, and seven genes involved in light response and anthocyanin synthesis were selected. Actin was used as an internal reference gene for gene expression correction, and the sequences of primers are listed in **Table 1**. qRT-PCR was performed in triplicate using a Roche LightCycler 480 fluorescent PCR system (Roche, Basel, Switzerland) and a MagicSYBR Mixture fluorescent dye kit (Kangwei Century Biotechnology Co., Ltd., Beijing, China) using the same programs and method as those used for structural gene expression.

### 2.6 Statistical analysis

The experimental data were processed using SPSS 22.0 (SPSS Inc, Chicago, USA), and Duncan’s test was performed by employing one-way ANOVA. The significance level was set at *p <* 0.05.

## 3 Results

### 3.1 Anthocyanin accumulation and expression of anthocyanin biosynthetic genes in purple pepper under blue light

**Figure 2:**
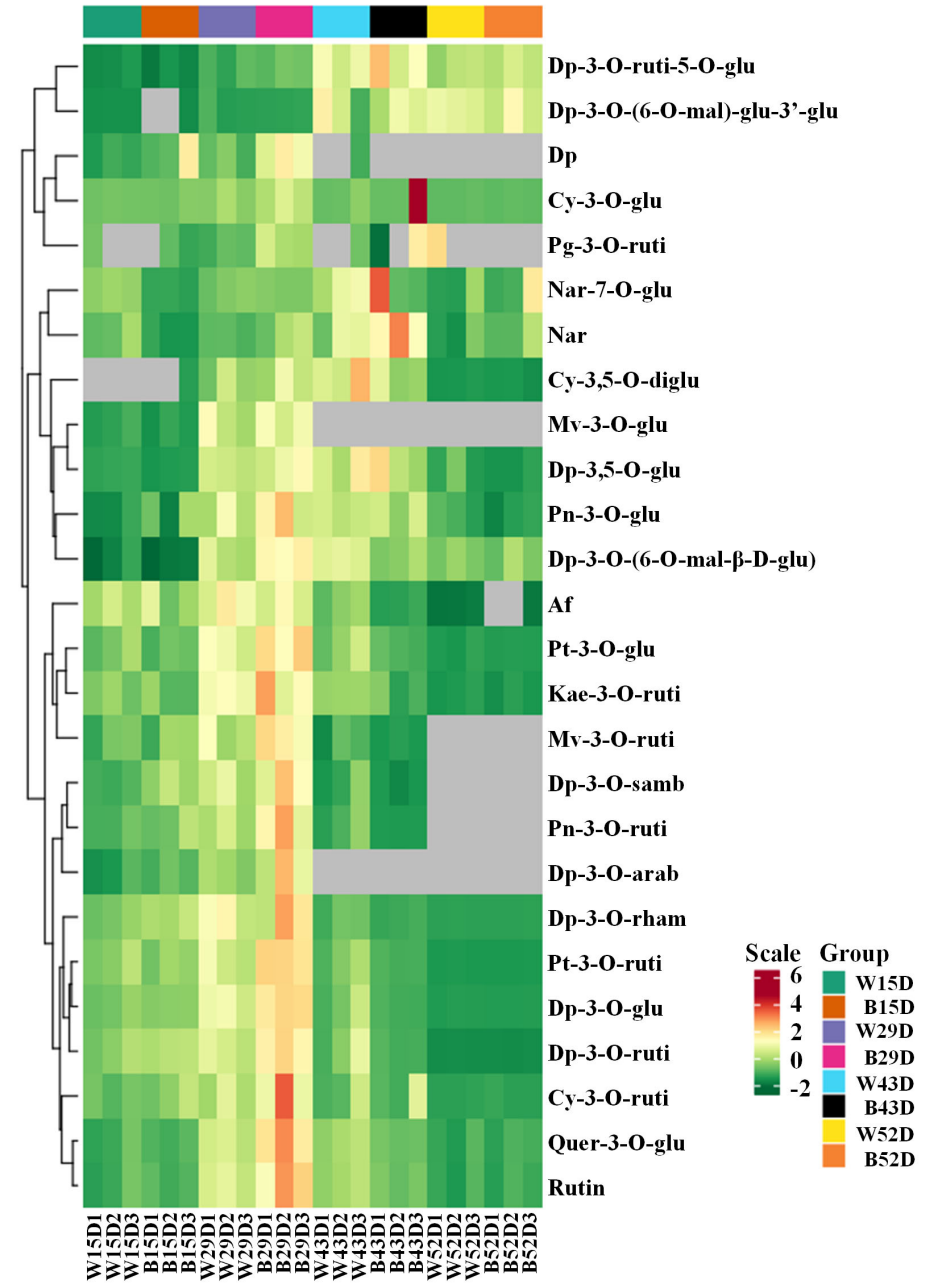
Cluster analysis of anthocyanins in the peel of purple pepper fruit at 15, 29, 43, and 52 DAF under white and blue light for 4 h. DAF, day after flowering; W, white light; B, blue light; Dp, delphinidin; Cy, cyanidin; Pg, pelargonidin; Nar, naringenin; Mv, malvidin; Pn, peonidin; Af, afzelin; Pt, petunidin; Kae, kaempferol; Quer, quercetin; Rutin, rutin; ruti, rutinoside; glu, glucoside; mal, malonyl; diglu, diglucoside; samb, sambubioside; arab, arabinoside; rham, rhamnoside.

**Figure 3:**
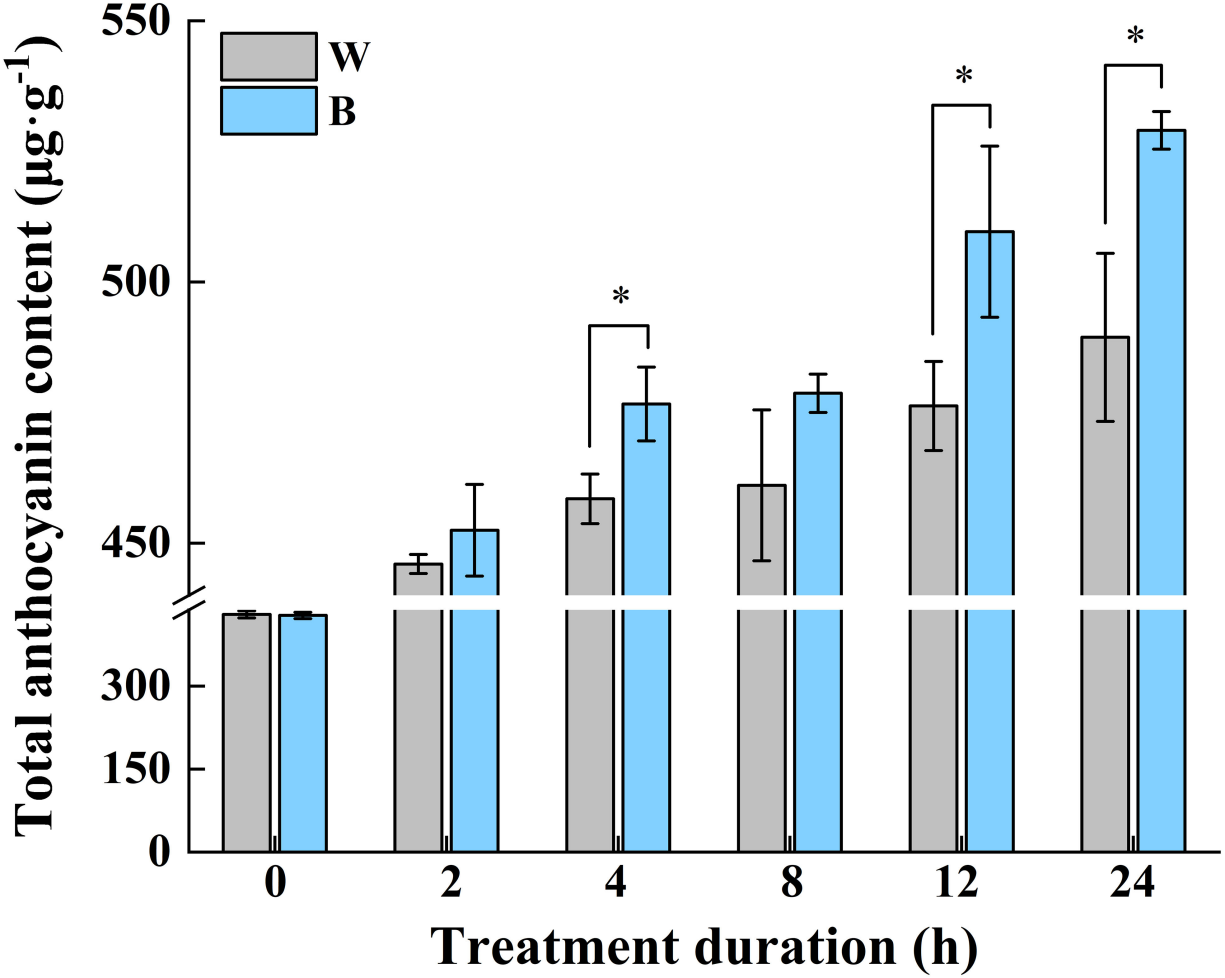
Changes of total anthocyanin content in purple pepper fruit at 29 DAF with treatment duration of 0 h, 2 h, 4 h, 8 h, 12 h, and 24 h under white and blue light. Anthocyanin levels are indicated as the mean ± SD of three biological replicates. DAF, day after flowering; W, white light; B, blue light. *p < 0.05.

**Figure 4:**
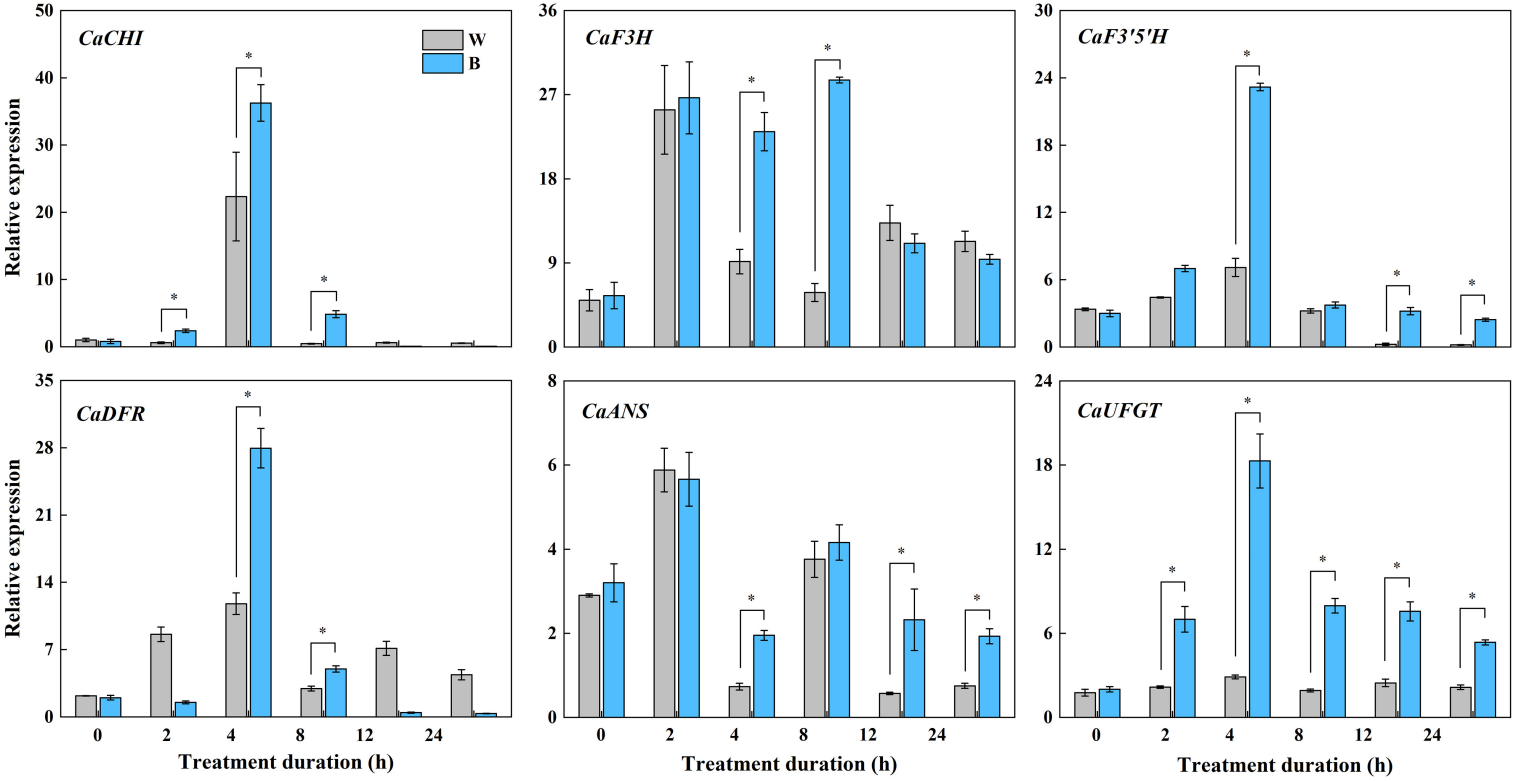
Expression variations of structural genes involved in anthocyanin synthesis in purple pepper fruit at 29 DAF with treatment duration of 0 h, 2 h, 4 h, 8 h, 12 h, and 24 h under white and blue light. Relative expression is indicated as the mean ± SD of three biological replicates. DAF, day after flowering; W, white light; B, blue light; CHI, chalconeisomerase; F3H, flavanone-3-hydroxylase; F3′5′H, flavonoid 3′,5′ hydroxylase; DFR, dihydroflavonol 4-reductase; ANS, anthocyanidin synthase; UFGT, UDP-glucose: flavonoid 3-O-glucosyltransferase. *p < 0.05.

In our previous study, the most abundant anthocyanin in pepper fruit at different ripening stages under white and blue light was identified as Dp glycosides, which accounted for 94.70% of the total anthocyanin content, followed by glycosides of petunidin (Pt), cyanidin (Cy), pelargonidin (Pg), peonidin, and malvidin (**Figure 2**). Moreover, these metabolites were found to be highly accumulated in fruit at 29 DAF. In this study, we determined the total anthocyanin content and structural genes involved in anthocyanin biosynthesis in pepper fruit at this ripening stage with treatment duration of 0 h, 2 h, 4 h, 8 h, 12 h, and 24 h. It was indicated that the total anthocyanin content increased over time under both spectrum treatment (**Figure 3**), and the level was significantly higher in fruit under blue light than that of fruit under white at 4 h, 12 h, and 24 h (*P* < 0.05). The transcript abundance of anthocyanin biosynthetic genes was also hardly affected (**Figure 4**). Compared with white light, the structural genes, especially *CaF3′5′H* and *CaUFGT*, were significantly upregulated at 4 h of blue-light treatment. This indicates that the genes related to anthocyanin synthesis in purple pepper are regulated by blue light, and a short period of blue-light irradiation can affect their expression levels.

**Figure 5:**
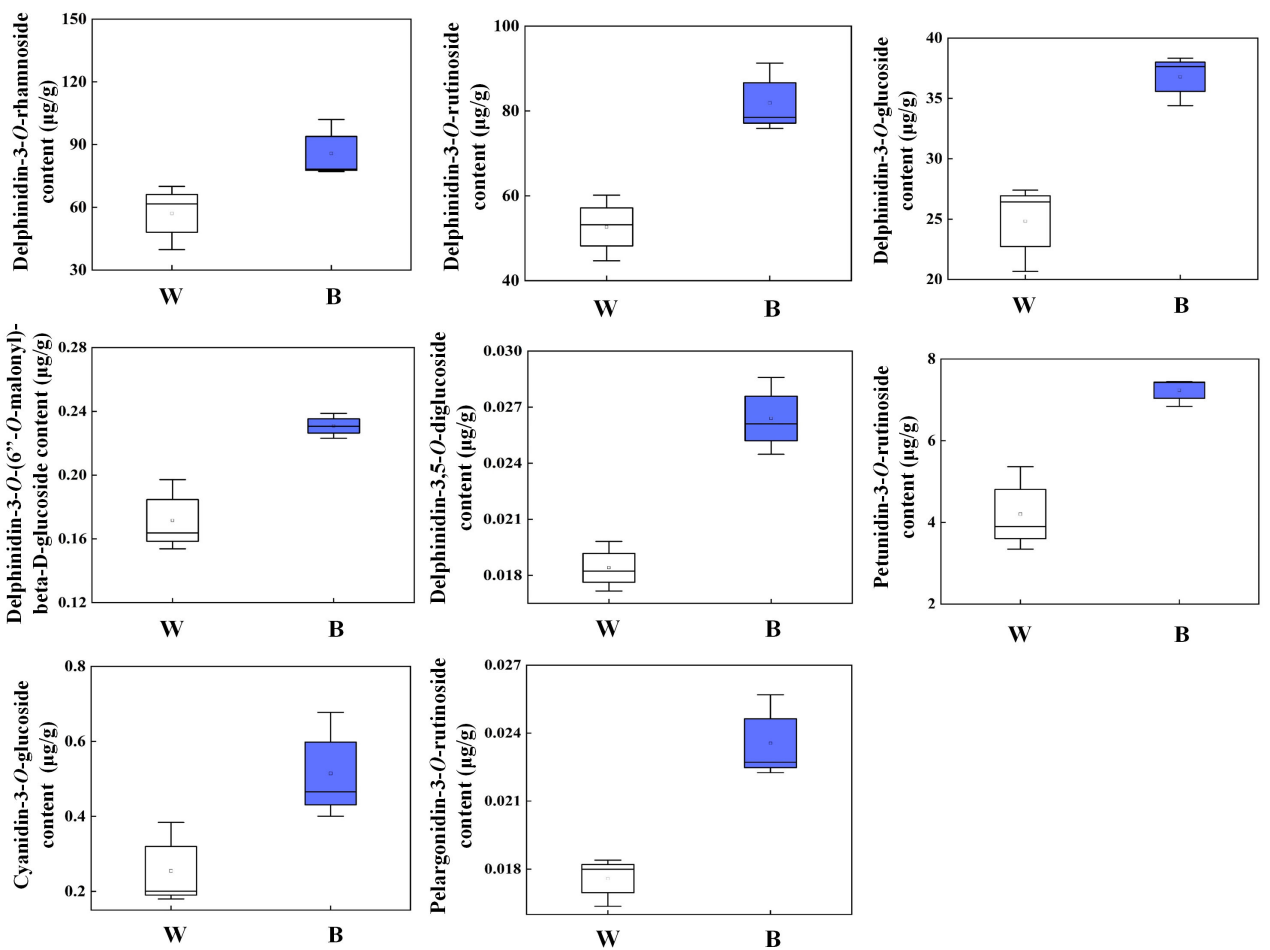
Different kinds of anthocyanin accumulation in purple pepper fruit at 29 DAF with 4 h of white and blue-light irradiation. Anthocyanin levels are indicated as the mean ± SD of three biological replicates. DAF, day after flowering; W, white light; B, blue light.

We thus investigated the levels of primary anthocyanins, including Dp, Pt, Cy, and Pg derivatives in pepper fruit at 29 DAF treated with 4 h of blue light in the present study (**Figure 5**). The Dp glycosides mainly consisted of Dp-3-*O*-rhamnoside, Dp-3-*O*-rutinoside, and Dp-3-*O*-glucoside, which were specifically accumulated in both blue- and white-light–irradiated pepper fruit than those of Dp-3-*O*-(6’’-*O*-malonyl)-beta-D-glucoside and Dp-3,5-*O*-diglucoside. The concentration of Pt-3-*O*-rutinoside, Cy-3-*O*-glucoside, and Pg-3-*O*-rutinoside was significantly lower relative to the Dp glycosides from fruit irradiated by both spectrum. Moreover, all these metabolites were evidently enhanced by blue light in comparison with white light (*P* < 0.05).

### 3.2 Summary of mRNA sequencing

**Figure 6:**
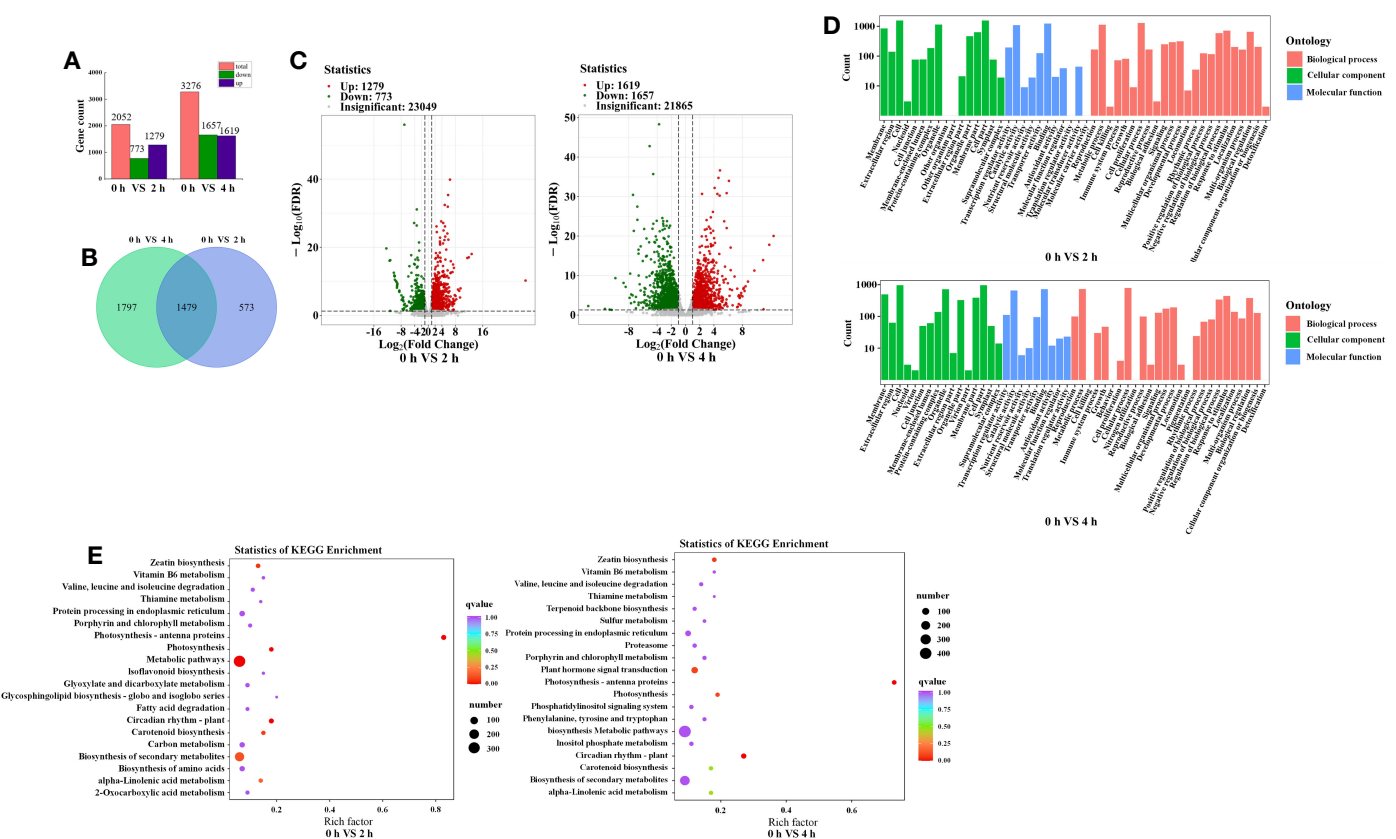
Comprehensive analysis of transcriptome data. **(A)** Number of DEGs identified by transcriptome sequencing analysis in purple pepper fruit with 2 h and 4 h of blue-light irradiation. **(B)** Venn diagram depicting numbers of DEGs. **(C)** Volcano plots of the DEGs between 0 h versus 2 h and 0 h versus 4 h. **(D)** GO enrichment analysis of the DEGs between 0 h versus 2 h and 0 h versus 4 h. **(E)** 20 tops of KEGG enrichment analysis of the DEGs between 0 h versus 2 h and 0 h versus 4 h. The ordinate represents KEGG pathway, and the abscissa represents rich factor. The larger the rich factor, the greater the degree of enrichment. The larger the point, the greater the number of differentially enriched genes in the pathway. The redder the dot, the more significant the enrichment. DEGs, differentially expressed genes; GO, Gene Ontology; KEGG, Kyoto Encyclopedia of Genes and Genomes.

To investigate the mechanism of anthocyanin synthesis regulated by blue light in postharvest purple peppers, mRNA sequencing was used to analyze the DEGs. Among the total nine sequencing samples, each sample produced 4.51-Gb clean reads on average. Q30 values were distributed between 93.23% and 94.14%. GC content was distributed between 41.17% and 42.11%. Mapping clean reads to the specified pepper reference genome (Pepper_Zunla_1_Ref_v1.0 version), the average mapping ratios of these sequencing samples were 94.12% (**Table 2**). This indicated that the selected reference genome was adequate for subsequent analysis. A total of 2,052 and 3,276 DEGs were screened, of which 1,279 and 1,619 upregulated genes as well as 773 and 1,657 downregulated genes were screened at 2 h and 4 h, respectively (**Figures 6A, C**). We obtained 3,276 DEGs from the samples and screened 1,479 genes (**Figure 6B**). Furthermore, the correlation was above 84% for all nine sequenced samples (**Supplementary Figure 1**).

**Table 2:** Quality evaluation of sample sequencing output data.

Sample	Reads Sum	≥ Q30 (%)	GC Content (%)	Mapped Ratio (%)
0h1	43,839,658	94.12	41.45	94.46
0h2	45,492,834	94.14	41.89	94.75
0h3	43,250,106	93.67	41.30	94.16
2h1	44,594,684	93.23	42.11	94.48
2h2	43,014,342	93.34	41.43	93.90
2h3	42,465,108	93.89	41.49	94.21
4h1	55,414,632	93.87	41.58	94.10
4h2	43,301,086	93.68	41.17	93.08
4h3	44,140,764	93.52	41.71	93.94
Average	45,057,023.78	93.72	41.57	94.12

Note reads sum, total number of paired-end reads in clean data; ≥ Q30, the percentage of bases with a clean data quality score of 30 or higher; GC content, the percentage of G and C bases in clean data with respect to the total bases; mapped ratio, the mapped to the reference genome reads as a percentage of total reads.

### 3.3 GO and KEGG functional enrichment analysis

Functional annotation of DEGs through the GO database identified 51 important functional categories, including biological process, molecular function, and cellular component. Compared with 0 h, the GO entries annotated for 2 h and 4 h of blue-light treatment were mainly for processes such as biosynthesis of secondary metabolites, stress response, transmembrane transport, and transcriptional regulatory activity (**Figure 6D**). As anthocyanins are secondary metabolites, they are important parts of the metabolic pathway. The anthocyanins were unstable and need to be transferred to the vesicles for storage. Therefore, anthocyanin accumulation is related to transporter protein activity. Second, in this study, we focused on blue-light–induced anthocyanin accumulation. The previous study indicated that the effect of light on anthocyanin synthesis involves light signal transduction and transcriptional regulation processes. In conclusion, we focused on the functional groups of metabolic processes (GO:0008152), signal transduction (GO:0023052), transcriptional regulatory activity (GO:0140110), transporter protein activity (GO:0005215), and stress response (GO:0050896).

The DEGs were used to make KEGG pathway enrichment analysis and were found to be significantly enriched in metabolic pathway (ko01100), secondary metabolite biosynthesis (ko01110), plant circadian movements (ko04712), and plant mitogen-activated protein kinase (MAPK) signaling pathways (ko04016) as well as phytohormone signaling (ko04075) (**Supplementary Figure 2**). Furthermore, 20 significantly different pathways were selected (**Figure 6E**). As important parts of environmental adaptation and biosynthesis of secondary metabolites, circadian rhythm pathway (ko04712), flavonoid biosynthesis pathway (ko00941), and anthocyanin biosynthesis pathway (ko00942) were selected, which were associated with light response and anthocyanin synthesis, respectively. Genes associated with light response and anthocyanin biosynthesis were screened against selected DEGs enriched for the KEGG pathway and combined with GO functional annotation. Twenty-four genes showed expressional differences in pathways of circadian rhythm, flavonoid biosynthesis, and anthocyanin biosynthesis, which contained three genes involved in light response and six structural genes associated with anthocyanin biosynthesis, such as *CaLHY*, *CaCHI*, *CaDFR*, *CaLDOX*, and *CaUFGT* (**Table 3**).

**Table 3:** The detailed information of DEGs related to anthocyanin biosynthesis.

Gene name	Gene ID	log_2_ FC	p-value	q-value
*CaCHI*	LOC107871144	−2.1375	2.66828E^−05^	0.00084
*CaDFR*	LOC107860031	−2.5558	0.00027	0.00549
*CaLDOX4*	LOC107845999	4.8146	9.35842E^−06^	0.00035
*CaLDOX3*	LOC107844833	4.2115	0.00055	0.00956
*CaFLS*	LOC107861925	1.489	0.00013	0.00311
*CaUFGT*	LOC107867844	2.5983	7.06399E^−05^	0.00188
*CaLHY*	LOC107845265	−3.3882	1.78769E^−35^	7.43917E^−32^
*CaAPRR5*	LOC107862999	2.527	5.00799E^−10^	6.01151E^−08^
*CaPRR37*	LOC107850219	1.498	1.5002E^−12^	3.09562E^−10^

DEGs, differentially expressed genes; FC, fold change.

### 3.4 The selection of TFs and validation of transcriptome sequencing by qRT-PCR

**Figure 7:**
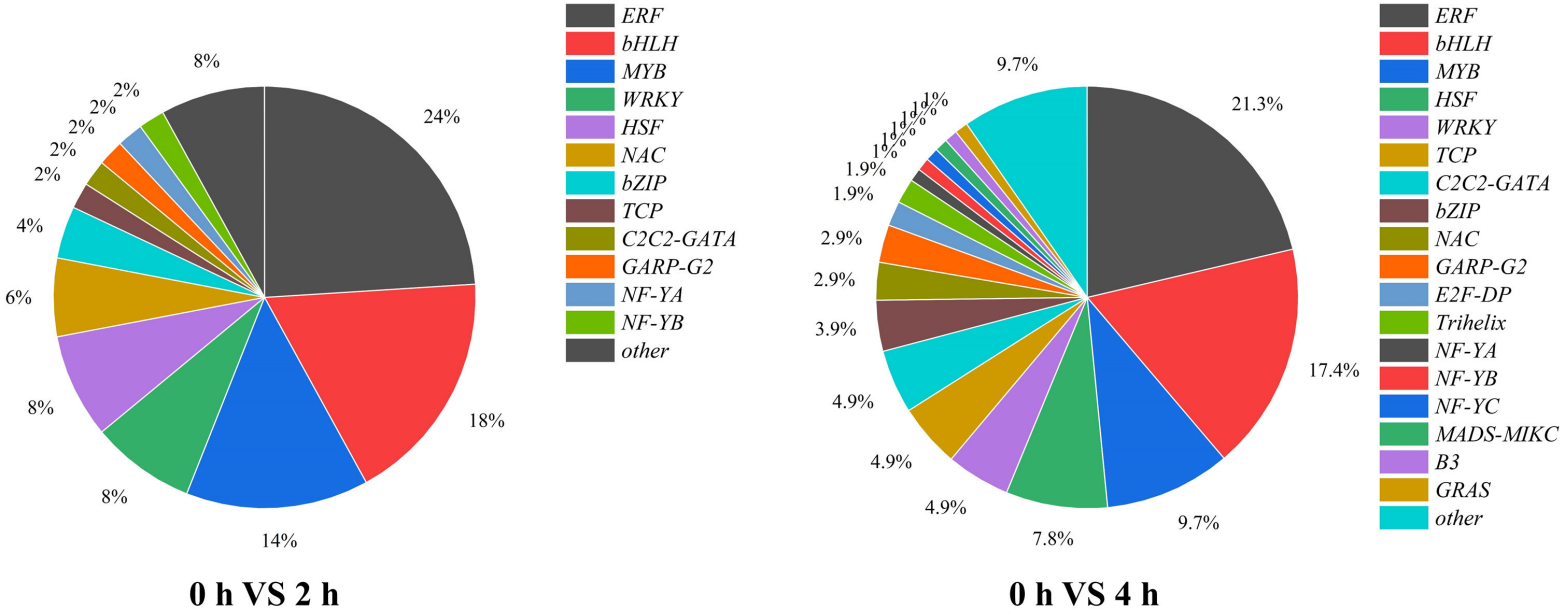
The proportion and types of transcription factors in purple pepper fruit with 2 h and 4 h of white and blue-light irradiation.

The expression of structural genes involved in anthocyanin biosynthesis is regulated by upstream TFs. Thus, TF associated with light signaling and anthocyanin synthesis must be existed. In our study, a total of 50 and 103 genes were defined as TFs for the 2 h and 4 h of treatments, respectively, which classified into 13 and 19 TF families, among which ERF family was the largest, followed by bHLHs and MYBs (**Figure 7**).

**Figure 8:**
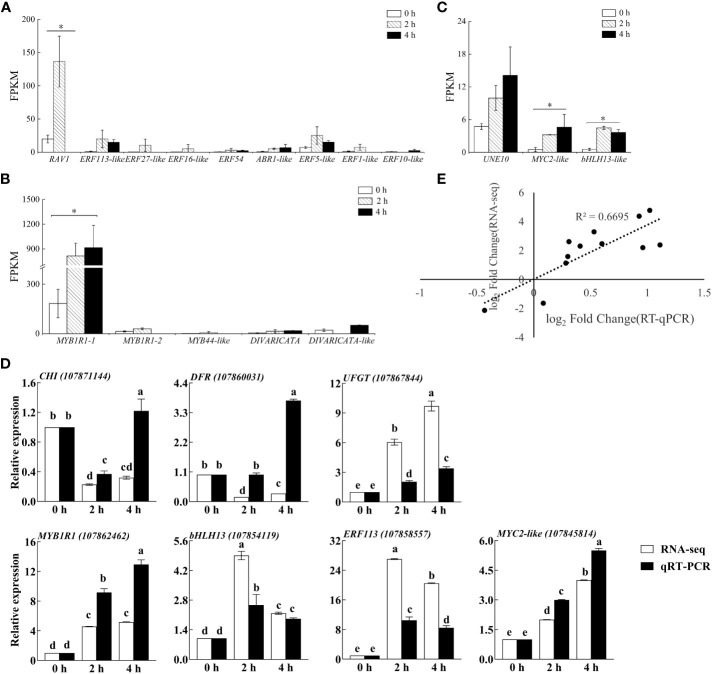
The FPKM verification of all transcription factors identified by transcriptome sequencing analysis in purple pepper fruit with 2 h and 4 h of white and blue-light irradiation, including families of **(A)** ERF, **(B)** MYB, and **(C)** bHLH. **(D)** Verification of relative expression levels of structural genes and transcription factors associated with the anthocyanin synthesis pathway in purple pepper fruit under 2 h and 4 h of white and blue-light irradiation. **(E)** Correlation analysis between transcript levels of qRT-PCR and transcriptome sequencing data. Relative expression is indicated as the mean ± SD of three biological replicates. FPKM, fragments per kilobase of transcript per million fragments mapped reads. Bars with different lowercase letters indicate significant differences at p < 0.05.

The gene functions derived from ERFs mainly in ethylene response and take participate in plant secondary metabolites biosynthesis. We found that eight and five genes were upregulated, and three and fifteen genes were downregulated from ERFs in purple pepper peel after 2 h and 4 h of exposure of blue light relative to 0 h, respectively. Comparison of FPKM values for upregulated genes revealed significant differences in *ERF113-like* (LOC107858557), *ABR1-like* (LOC107867626), *ERF54* (LOC107855040), and *ERF5-like* (LOC107871072) at both 2 h and 4 h of treatments (**Figure 8A**). However, *RAV1* (LOC107847951), *ERF27-like* (LOC107851517), *ERF16-like* (LOC107874934), and *ERF1-like* (LOC107872603) were significantly highly expressed only at the treatment of 2 h.

Many previous studies have been reported on the involvement of the MYB family in plant anthocyanin biosynthesis. In the present study, we found four upregulated genes and three downregulated genes from MYBs in purple pepper peel after 2 h and 4 h of irradiation under blue light relative to 0 h. Then, four upregulated MYB genes, including *MYB1R1-1* (LOC107862462), *MYB1R1-2* (LOC107868015), *MYB44-like* (LOC107869439), and *DIVARICATA* (LOC107862150) were further screened for analysis (**Figure 8B**). Among them, *MYB1R1-1* (LOC107862462) accumulated specifically under 2 h and 4 h of blue spectrum irradiation and was defined as *CaMYB1R1*. The expression level of *MYB44-like* (LOC107869439) was significantly upregulated under 2 h irradiation of blue light compared with 0 h, but there was almost no expression under 4 h of treatment.

bHLH family TFs are also involved in anthocyanin biosynthesis. We found that eight DEGs in purple peppers under both 2 and 4 h of blue-light irradiation compared with white light. Among them, five genes were upregulated and three genes were downregulated. Further screening analysis of significant differences in expression levels of *UNE10* (LOC107845481), *MYC2-like* (LOC107845814), and *bHLH13-like* (LOC107854119) suggested that they were all significantly elevated under 2 h and 4 h of treatment (**Figure 8C**).

To confirm the reliability of RNA-seq data, three structural genes and four TFs were chosen, and their relative expression profiles were quantified using qRT-PCR (**Figure 8D**). The expression of the selected genes in transcriptome sequencing FPKM (log_2_fold change) was correlated with the relative gene expression level log_2_ (2^−ΔΔCt^) values in qRT-PCR. The expression patterns of these genes obtained from qRT-PCR were highly consistent with those in the RNA-seq data (**Figure 8E**), suggesting that the results of RNA-seq were reliable.

## 4 Discussion

Anthocyanins are usually considered as a cause of purple pepper’s color (Tang et al., 2020; Meng et al., 2022). It was reported that six common anthocyanins have been found and Dp derivatives are the predominant anthocyanins in purple *Solanaceous* vegetables (Liu et al., 2018b), specifically accumulated in purple pepper fruit (Meng et al., 2022; Wang et al., 2022b). In this study, the accumulation levels of these Dp glycosides particularly Dp-3-*O*-rhamnoside, Dp-3-*O*-rutinoside, and Dp-3-*O*-glucoside were positively correlated with the purple peel coloration, implying that these Dp derivatives mainly contribute to the purple color of pepper fruit (Liu et al., 2020). Furthermore, these compounds were most responsive to the blue-light irradiation relative to other Dp derivatives as well as the Pt, Cy, and Pg derivatives in comparison with white light, indicating that blue light mainly accelerates the postharvest fruit of purple pepper to appear purple-black color by promoting the accumulation of Dp-3-*O*-rhamnoside, Dp-3-*O*-rutinoside, and Dp-3-*O*-glucoside.

Existing findings reported that the transcript level of LBGs, such as *F3′5′H* and *UFGT*, coincides well with anthocyanin content and is significantly higher in pigmented compared with non-pigmented tissues, suggesting that variations in LBG expression determine the quantitative variation of anthocyanins in *Solanaceous* vegetables (Liu et al., 2018b). This was confirmed in our present study. Moreover, our validation results suggested that, compared with white light, the anthocyanin biosynthetic LBG, such as *CaUFGT* (**Figure 8D**), which is a key link in the formation of anthocyanin diversity, was separately regulated and highly responsive to blue light at both 2 h and 4 h of treatment. This led to the higher accumulation of anthocyanin exposed to blue spectrum compared with control fruits. Furthermore, the expression level of *CaF3’5’H*, the gene directing dihydroflavonol precursors to Dp compounds biosynthesis, was found to be highly upregulated under this wavelength (**Figure 4**). These changed the anthocyanin profile toward Dp derivatives in purple peppers under blue light (Samkumar et al., 2021).

In addition to structural genes, TFs, such as MYB, bHLH, and ERF families, are also the key factors in the regulation of anthocyanin metabolism in plants (Meng et al., 2022; Sun et al., 2023). A high number of DEGs representing ERF, MYB, and bHLH genes were found from our RNA-seq libraries, among which the expression levels of *CaMYC2*-*like* and *CaERF113* under both 2 h and 4 h of blue-light irradiation were validated through qRT-PCR, indicating that their regulation is strongly influenced by this light spectral quality. As a jasmonic acid (JA) signaling hub, MYC2 participates in anthocyanin synthesis in many crops (Li et al., 2018; Liu et al., 2022a). However, unlike the previous study in which MYC2 is a negative regulator of blue-light–induced photomorphogenesis (Yadav et al., 2005). *CaMYC2*-*like* was positively expressed correlated with variation tendency of key anthocyanin biosynthetic genes under 2 h and 4 h of blue-light spectrum treatment compared with white light in our present study, and this indicated its important role in anthocyanin biosynthetic pathway regulated by blue light (Liu et al., 2019).

It has been previously reported that *ERF* genes functioned as a transcriptional activator in promoting anthocyanin biosynthesis by interacting with related *MYB* genes to form the ERF-MYB protein complex or activating the transcription of anthocyanin biosynthetic genes, including *UFGT* (An et al., 2018; Chang et al., 2023). Furthermore, previous studies reported that the expression of *MYC2* was induced by JA in apple and activates the expression of *ACS1*, by upregulating the transcription of *ERF*, thereby promoting ethylene synthesis and fruit ripening (Li et al., 2017; Wang et al., 2022a). Thus, we speculated that CaMYC2-like was capable of combining directly with the *CaERF113* promoter to promote *CaACS1* transcription and facilitate pepper fruit ripening and coloring. On the other hand, CaERF113 could activate the promoters of downstream genes in the anthocyanin biosynthetic pathway of *CaUFGT*, ultimately accelerating anthocyanin accumulation in purple pepper fruit.

**Figure 9:**
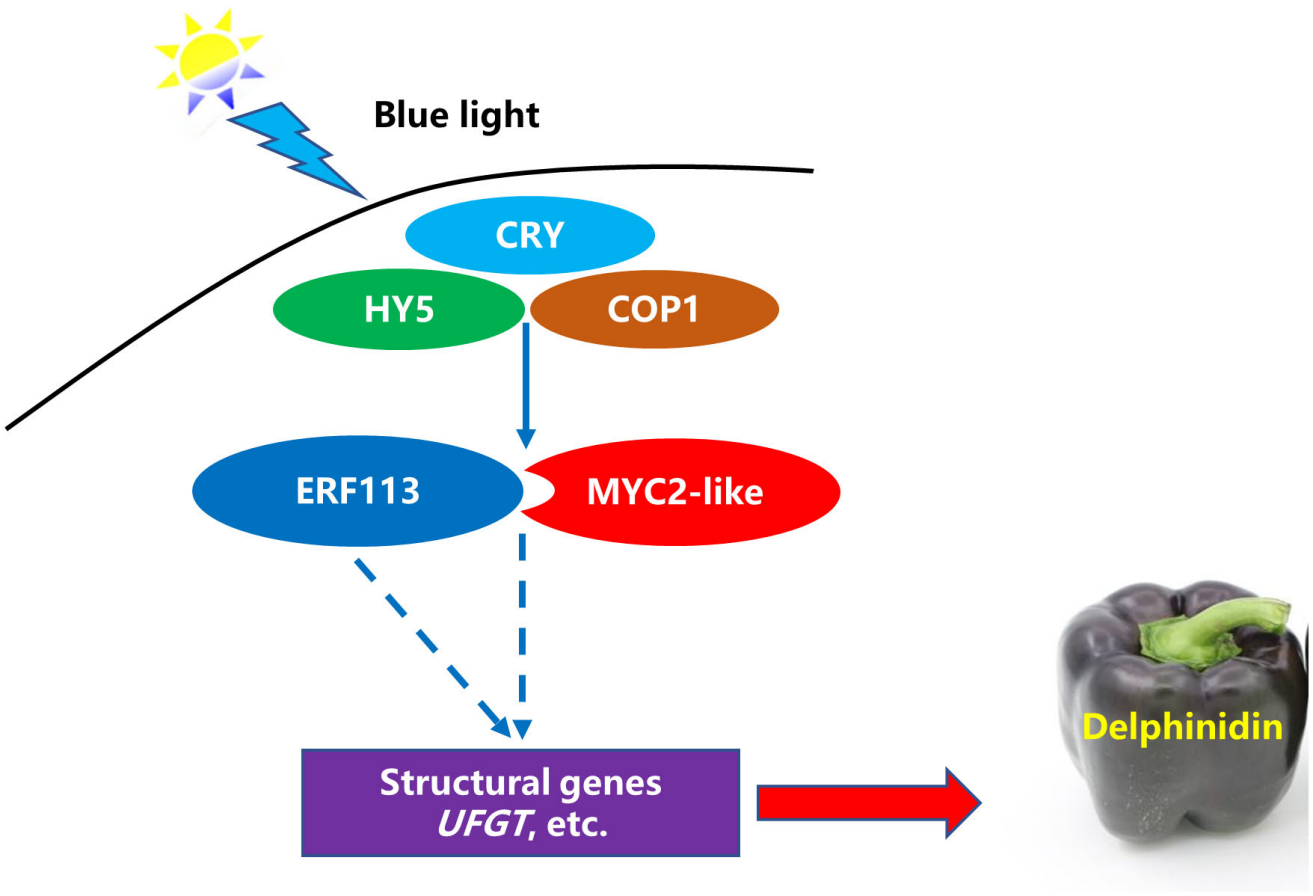
A possible model for blue-light–induced delphinidin derivatives biosynthesis in purple pepper fruit. The solid line indicates that the functional mechanism is clear, and the dotted line refers to the functional mechanism needs to be further studied.

MYBs commonly combine with bHLHs to form a protein complex that participates in the regulation of anthocyanin biosynthesis. Previous study found that MYB can interact with bHLH to promote the activity of the *UFGT* promoter and anthocyanin biosynthesis (Starkevic et al., 2020). In this research, *CaMYB1R1* was differentially expressed under blue light from RNA-seq analysis. However, the qRT-PCR results of this gene were inconsistent with the RNA-seq analysis. Whether it acts as a major TF, which is influenced by blue light and subsequently contributing to the anthocyanin biosynthesis, needs further investigation. CRY is an important photoreceptor and can induce anthocyanin synthesis under blue light (Sun et al., 2020). Previous studies have reported the involvement of an indirect action module CRY-COP1-HY5 in photomorphogenesis (Tao et al., 2018; Wang et al., 2020). Therefore, we inferred that blue-light–CRY system plays important roles in regulation of anthocyanin synthesis in purple pepper. On the basis of our results, we conclude that CaMYC2-like facilitates the promotion of Dp glycosides accumulation by interacting with CaERF113 and increasing *CaUFGT* expression under blue light (**Figure 9**).

## 5 Conclusion

Transcriptome data were applied to illustrate the regulation metabolism of blue light on anthocyanin biosynthesis metabolic pathway in purple pepper fruit. Changes in anthocyanin metabolites, especially Dp glycosides, comprising Dp-3-*O*-rhamnoside, Dp-3-*O*-rutinoside, and Dp-3-*O*-glucoside, could be mainly responsible for the purple color. In addition, 12 TFs were also found to be potential contributors to anthocyanin metabolite biosynthesis regulated by blue light in pepper fruit. Correlation analysis of the changes in gene expression and transcriptome data suggested that blue-light spectrum could induce *CaMYC2-like* and *CaERF113* expression to promote the accumulation of Dp glycosides. These results provided valuable information on the anthocyanin metabolites and the candidate genes involved in the anthocyanin biosynthesis pathways in pepper.

## Data availability statement

The datasets presented in this study can be found in online repositories. The original data is publicly available at NCBI, PRJNA1014603.

## Author contributions

JG: Conceptualization, Data curation, Investigation, Visualization, Writing – original draft. YD: Investigation, Methodology, Project administration, Software, Writing – original draft. XW: Investigation, Methodology, Project administration, Software, Writing – original draft. DZ: Validation, Writing – review & editing. MW: Validation, Writing – review & editing. YL: Conceptualization, Formal Analysis, Funding acquisition, Methodology, Resources, Supervision, Writing – original draft, Writing – review & editing.

## Supplementary material

Supplementary Figure 1 Correlation chart of DEGs identified by transcriptome sequencing analysis in purple pepper fruit with 2 h and 4 h of white and blue-light irradiation. DEGs, differentially expressed genes.

Supplementary Figure 2 KEGG enrichment analysis of the DEGs between 0 h versus 2 h and 0 h versus 4 h. KEGG, Kyoto Encyclopedia of Genes and Genomes; DEGs, differentially expressed genes.
