# Supplementary figures and images for: Transcriptome analysis reveals the mechanism for blue-light–induced biosynthesis of delphinidin derivatives in harvested purple pepper fruit
Source: Front Plant Sci. 2023 Oct 26;14:1289120. doi: 10.3389/fpls.2023.1289120 (PMC10640979; doi:10.3389/fpls.2023.1289120)

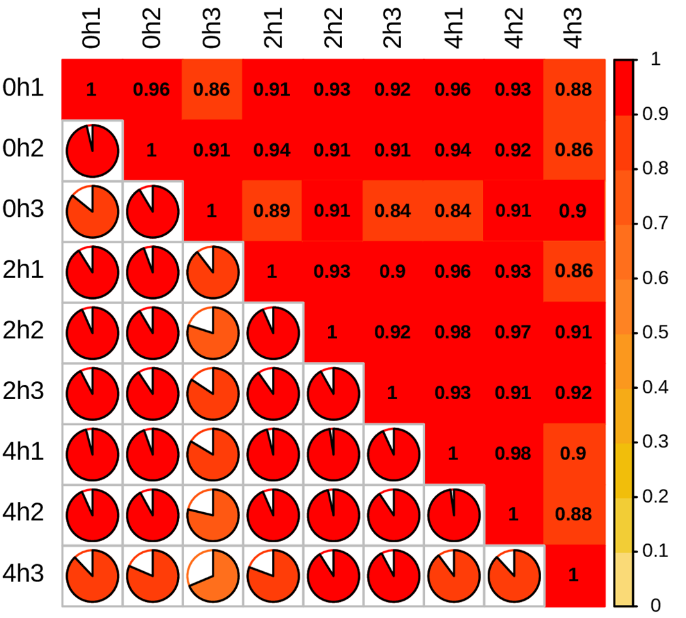

Supplement: Supplementary Figure 1 — Correlation chart of DEGs identified by transcriptome sequencing analysis in purple pepper fruit with 2 h and 4 h of white and blue-light irradiation. DEGs, differentially expressed genes. [file Image_1.tif]

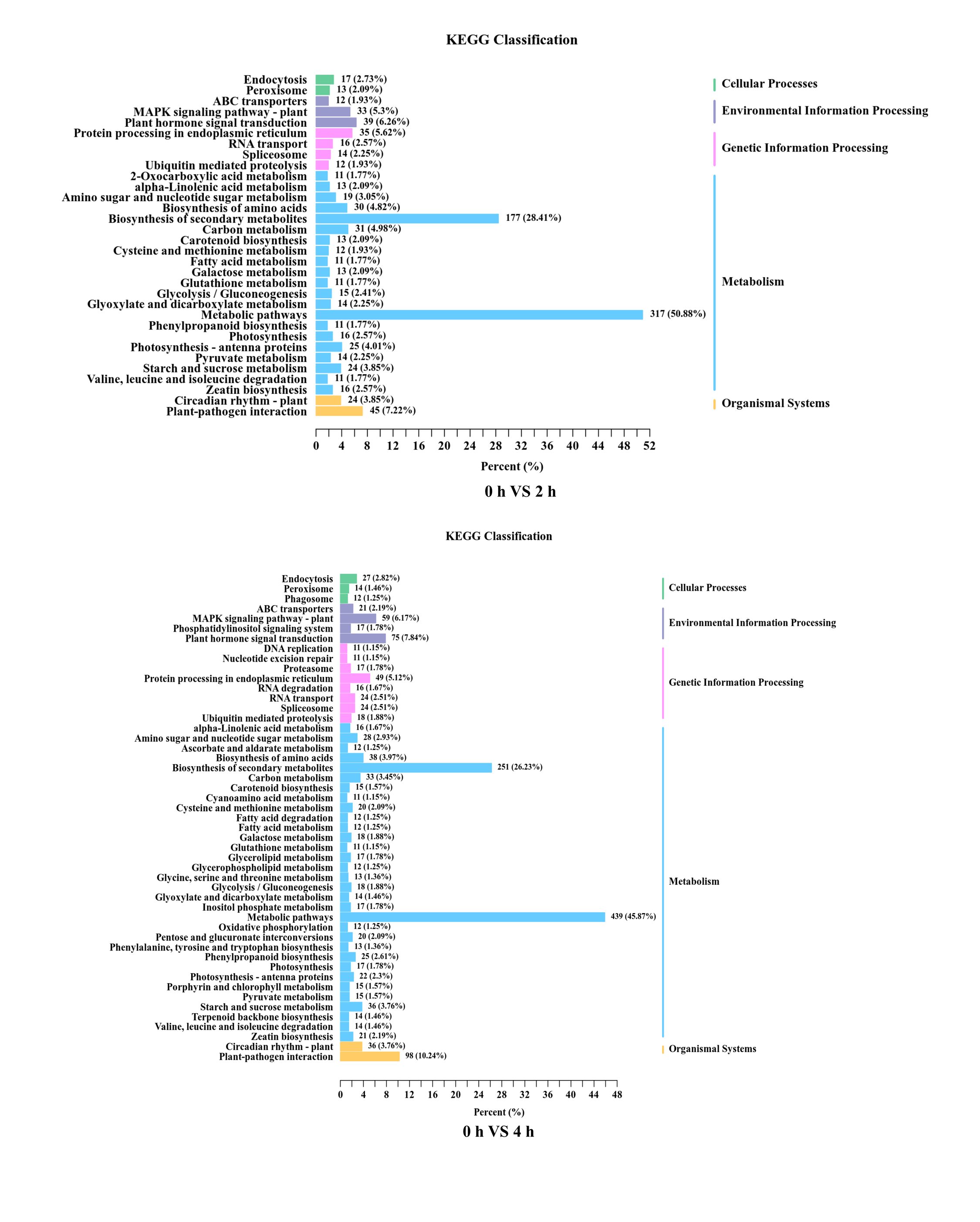

Supplement: Supplementary Figure 2 — KEGG enrichment analysis of the DEGs between 0 h versus 2 h and 0 h versus 4 h. KEGG, Kyoto Encyclopedia of Genes and Genomes; DEGs, differentially expressed genes. [file Image_2.tif]
